# Ceramide Metabolism Enzymes—Therapeutic Targets against Cancer

**DOI:** 10.3390/medicina57070729

**Published:** 2021-07-19

**Authors:** Ana Gomez-Larrauri, Upasana Das Adhikari, Marta Aramburu-Nuñez, Antía Custodia, Alberto Ouro

**Affiliations:** 1 Department of Biochemistry and Molecular Biology, Faculty of Science and Technology, University of the Basque Country, P.O. Box 644, 48980 Bilbao, Spain; ana.gomezlarrauri@osakidetza.eus; 2 Respiratory Department, Cruces University Hospital, P.O. Box 644, 48903 Barakaldo, Spain; 3 Ragon Institute of MGH, MITHarvard and HarvardMIT, Cambridge, MA 02139, USA; upasana.da@gmail.com; 4 Harvard Medical School, Boston, MA 02114, USA; 5 Clinical Neurosciences Research Laboratories, Health Research Institute of Santiago de Compostela (IDIS), Travesa da Choupana s/n, 15706 Santiago de Compostela, Spain; marta.aramburu.nunez@sergas.es (M.A.-N.); antia.custodia.malvido@sergas.es (A.C.)

**Keywords:** ceramide (Cer), sphingolipids (Sphs), cancer, ceramide 1-phosphate (C1P), shingosine 1-phosphate (S1P), deoxy-sphingolipids, apoptosis, cell proliferation

## Abstract

Sphingolipids are both structural molecules that are essential for cell architecture and second messengers that are involved in numerous cell functions. Ceramide is the central hub of sphingolipid metabolism. In addition to being the precursor of complex sphingolipids, ceramides induce cell cycle arrest and promote cell death and inflammation. At least some of the enzymes involved in the regulation of sphingolipid metabolism are altered in carcinogenesis, and some are targets for anticancer drugs. A number of scientific reports have shown how alterations in sphingolipid pools can affect cell proliferation, survival and migration. Determination of sphingolipid levels and the regulation of the enzymes that are implicated in their metabolism is a key factor for developing novel therapeutic strategies or improving conventional therapies. The present review highlights the importance of bioactive sphingolipids and their regulatory enzymes as targets for therapeutic interventions with especial emphasis in carcinogenesis and cancer dissemination.

## 1. Introduction

Sphingolipids are fundamental components of cell membranes. They were discovered in brain extracts by J. L. W. Thudichum in 1876 and were considered merely as structural molecules of cell architecture. It was not until 1986 when Hannun and co-workers demonstrated that some sphingolipids were bioactive molecules, showing that sphingosine was capable of inhibiting protein kinase C (PKC) [1]. Since then, many sphingolipids have been shown to play critical roles in cell activation and the control of cell and tissue homeostasis [2,3,4]. Among all of the sphingolipids that are present in the organism, sphingosine and ceramide, together with their phosphorylated forms sphingosine-1-phosphate (S1P) and ceramide-1-phosphate (C1P), are crucial regulators of cell physiology and pathology [5,6].

**Figure 1:**
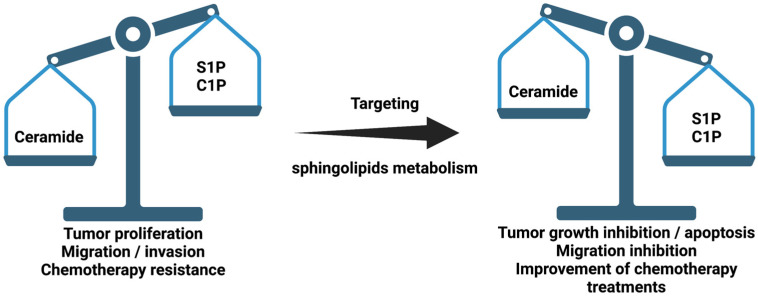
Schematic representation of sphingolipid ratio and their relationship with cancer. Sphingosine 1-phosphate (S1P) and ceramide 1-phosphate (C1P).

So far, more than 30 enzymes involved in sphingolipid metabolism have been characterized. These enzymes are highly regulated and are involved in the regulation of relevant pathophysiologic processes. Dysfunction of these enzymes may cause decompensation of the concentrations of their sphingolipid products, leading to a loss of cellular homeostasis. An important aspect that demonstrates the specificity and importance of sphingolipids in cell physiology is that small variations in sphingolipid concentrations can lead to or be a consequence of disease [4]. Among the most important sphingolipids are ceramide (Cer), sphingosine (Sph) and their phosphorylated forms, C1P and S1P. The intracellular concentrations of these metabolites define a precise balance that eventually determines cellular actions and fate. Whilst Cer and Sph are proapoptotic, C1P and S1P are proliferative and anti-apoptotic signals [4]. Sph is the precursor of S1P through phosphorylation by sphingosine kinases 1 and 2 (SphK1 and SphK2) [7,8], whereas C1P is produced by the action of CerK. Whether CerK exists as a sole gene product or as a family of different isoforms awaits further investigation. CerK resides in the Golgi apparatus, where it can phosphorylate ceramide that is transported by ceramide transfer protein (CERT) from the ER to the Golgi. Noteworthily, CerK-generated C1P can be transported by a recently identified C1P transfer protein (CPTP) from the Golgi to the plasma membrane, where it might participate in signal transduction processes [9]. Although CerK has also been identified in plants [10], it is the only enzyme capable of generating C1P in mammalian cells [11]. As mentioned above, an appropriate equilibrium between sphingosine and ceramide, versus their phosphorylated forms, can determine cell function and fate (Figure 1). When this balance is disturbed or altered, disease may arise. In particular, many inflammatory processes and pathologies, including cardiovascular diseases, neurodegenerative disorders, lung inflammatory disorders (such as chronic obstructive pulmonary disease (COPD) or asthma) type 2 diabetes or cancer, are characterized by disruption of sphingolipid homeostasis [12,13,14,15,16,17,18,19,20,21]. Moreover, it has been reported that treatment with short-chain Cer anologs is an effective treatment against certain cancers [22,23,24]. In addition, deoxy-sphingolipids have been shown to be of great importance when observing their potential as regulators of sphingolipid metabolism.

## 2. Sphingolipid Metabolism

**Figure 2:**
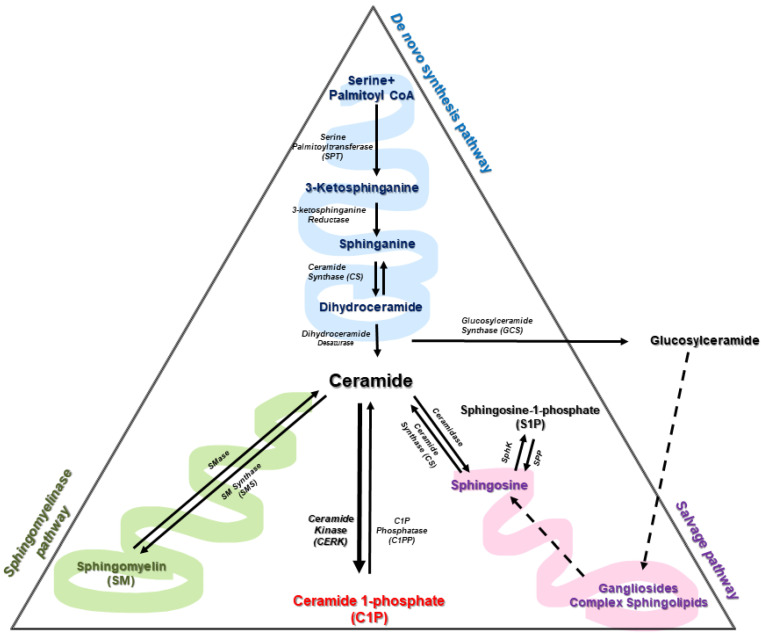
Representation of sphingolipid metabolism with ceramide as the central metabolite. SphK = sphingosine kinase; SPP = sphingosine-1-phosphate phosphatase and SMase = sphingomyelinase.

Cer is considered the central hub of sphingolipid metabolism. It has a dual role in cell biology as it can act as a precursor of complex sphingolipids or as second messengers to regulate a variety of physiologic and pathologic processes. In particular, it is well established that ceramides can induce cell cycle arrest and are potent inducers of apoptotic cell death [3,25]. As mentioned above, there are more than 30 enzymes involved in Cer metabolism, with a high degree of conservation and strict regulation. Ceramides can be mainly synthesized by three different pathways (Figure 2). 

The de novo pathway occurs integrally in the endoplasmic reticulum; it begins with the action of serine palmitoyl transferase (SPT) acting on palmitate and serine to form 3-ketosphinganine (or 3-ketodihydrosphingosine). The SPT complex is constituted by SPTLC1, SPTLC2, ssSPTa/b (two small subunits that enhance enzyme activity and also specify the acyl-CoA substrate) and four regulatory subunits, called ORMDLs (homologs of the yeast and plant Orms) [26]. Interestingly, recent studies have shown the efficiency of oral SPT inhibitors as anticancer drugs [27,28]. 3-ketodihydrosphingosine is then acted upon by 3-keto-dihydrosphingosine reductase (KDR), producing sphinganine. Ceramide synthase (CerS) can then catalyze the formation of dihydroceramides (dhCer) through the incorporation of acyl-CoA of different chain lengths. The last step of this pathway is catalyzed by a desaturase (Des1), which will introduce a trans double bond in position 4–5 (Δ4E) of dhCer to produce ceramide. In particular, specific deletion of this gene and polymorphisms are associated with the reduction of hepatic steatosis, attenuation of insulin and cognitive impairment in schizophrenia and with hypomyelination, leukodystrophy and degeneration of nervous system [29,30,31,32,33]. Of interest, the fatty acid desaturase family protein FADS3 has recently been described as a ceramide desaturase that adds a double bond at the Δ14Z position [34,35]. This enzyme could be of great importance in diseases related to the functional loss of Des1.

The sphingomyelinase (SMase) pathway is a catabolic pathway involving the degradation of sphingomyelin (SM) by the action of sphingomyelinases at the plasma membrane of cells, thereby generating Cer and phosphocholine. Cer formation through this pathway is considerably rapid compared to Cer synthesized de novo. Three major types of SMase exist, and these have been defined according to their optimal pH. These include lysosomal and plasma membrane acid SMase (aSMase), endoplasmic reticulum/nucleus and plasma membrane neutral SMase (nSMase) and alkaline SMase (alk-SMase), the latter being present in the intestinal track and human bile [36,37]. aSMase and nSMase are involved in cell signaling processes, whereas alkaline SMase participates in the digestion of SM incorporated in the diet. Of note, some pro-inflammatory cytokines, such as tumor necrosis factor α (TNF-α) or interleukin-1β (IL-1β), stimulate SMase activity to increase Cer concentration in cells [38,39,40]. Furthermore, both aSMase and nSMase activities can be stimulated by some anticancer drugs as part of their mechanism of action and by irradiation of cells with ultraviolet (UV) or ionizing radiation [41].

The salvage pathway is also a catabolic pathway. It involves the degradation of complex sphingolipids in acidic compartments, such as lysosomes, through a series of enzymatic reactions that give rise to Cer. The last step comprises the conversion of glucosylceramide by acid β-glucosidase 1 (β-GCase) to Cer. Dysfunction or deficiency of this enzyme leads to the development of lysosomal storage diseases (LSDs) [20].

The Cer is then transported to the cytosol, where ceramide synthases (CerS), which are located in the ER, catalyze the incorporation of acyl-CoAs to form Cer [42]. To date, six different CerS have been described, and these are encoded by six different genes. These enzymes vary in their levels of expression, cell type and their affinity for hydrocarbon chains of different lengths [43]. CerS enzymes have been implicated in different pathologies, chemotherapy resistance and oxidative stress [44,45,46,47,48]. Therefore, CerSs are enzymes also under study for therapeutic use [19]. There are different described enzymes, such as alkaline ceramidases (encoded by ACER1, ACER2 and ACER3), acidic ceramidase (encoded by ASAH1) and neutral ceramidase (encoded by ASAH2) [49,50,51,52]. ASAH1 is ubiquitously located in lysosomal compartments, while ASAH2 is expressed in plasma membranes and mainly in the small intestine and colon [49]. 

One of the most important aspects in cell homeostasis is the balance between the species of Cer, C1P and S1P, which will determine cell fate. In addition to the above pathways, Cer can be interconverted into C1P or S1P by different enzymatic activities.

Sphingosine Kinase (SphK)/S1P phosphatase. A relevant aspect of Sph metabolism is that it can be phosphorylated by sphingosine kinases to form S1P, which is bioactive and can control many physiologic and pathologic processes. Dephosphorylation of S1P to Sph is carried out by specific S1P phosphatases (SPP) or by lipid phosphate phosphatase (LPP) activity (reviewed by Gangoiti et al. [3]), whereas complete breakdown to non-sphingolipid moieties (hexadecenal and phosphoethanolamine) is catalyzed by S1P lyase [53].

CerK and C1P phosphatases. C1P levels are mainly regulated by the actions of Cerk and ceramide phosphate phosphatases (CPP) [54], less specific lipid phosphate phosphateases (LPPs) can also efficiently degrade C1P [55]. Once synthesized, Cer can be transported to Golgi apparatus by ceramide transfer protein (CERT) where it can be phosphorylated by Cerk Moreover, sphingomyelinase phosphodiesterase like 3b (*SMPDL3b*), an enzyme with high homology to aSMase that is present in podocytes, can also modulate C1P levels. Podocytes are important components of the renal glomeruli, preventing the leakage of plasma proteins into the urine [56]. This enzyme impedes the access of Cerk to its ceramide substrate thereby also reducing intracellular C1P concentrations [57].

## 3. Enzymes of Ceramide Metabolism

### 3.1. The De Novo Pathway of Ceramide Synthesis

#### 3.1.1. Serine Palmitoyl Transferase (SPT)

Serine palmitoyl transferase (SPT) is a heteromeric enzyme, composed of three subunits, that catalyzes the first step of the de novo ceramide synthesis pathway. The protein is localized to the endoplasmic reticulum. It catalyzes the condensation of L-serine and palmitoyl-coenzyme A to generate 3-keto-sphinganine (Figure 1). Several studies have described an increase in SPT activity in response to chemotherapy and radiotherapy in various types of cancer. So far, some SPT inhibitors that block tumor growth have been described. In particular, myriocin (also known as ISP-1) is a potent SPT inhibitor [58]. It was discovered as an antifungal antibiotic from *Myriococcum albomyces* [59], and later it was shown to block SPT [60]. Treatment with myriocin caused growth inhibition of breast cancer [19] and B16F10 melanoma cells through G2/M phase arrest [61]. In addition, there is a positive correlation between SPT enzyme inhibition and growth suppression of a human lung adenocarcinoma cell line (HCC4006) [62]. Furthermore, inhibition of SPT with myriocin or knocking down the enzyme expression with a specific siRNA inhibited the proliferation of human U87MG glioblastoma cells, an action that was associated with suppression of intracellular S1P levels [63]. Synthetic molecules of imidazopyridine and pyrazolopiperidine derivatives also showed high affinity and inhibitory capacity for SPT, decreasing ceramides levels in serum by 50% in mice that were treated orally for one week, and they were proposed as potential treatments for diabetes [28]. The authors of the latter report also designed two new inhibitors of SPT, tetrahydropyrazolopyridine and 3-phenylpiperidine. Both of these compounds were able to reduce ceramide concentration in the blood, and they inhibited the proliferation of HCC4006 and PL-21 cells derived from an acute promyelocytic leukemia [27,64].

It has been proposed that the antitumor activity of SPT is caused by increasing the levels of proapoptotic ceramides. In fact, some anticancer drugs exert at least part of their therapeutic effects through the activation of SPT. In particular, fenretinide, a synthetic retinoid and cytotoxic molecule to a variety of cancer cells, increased the concentration of dhCer and Cer, leading to neuroblastoma cell apoptosis, an action that was associated with the interaction of this drug with SPT and DeS1 [65]. Furthermore, a fenretinide metabolite, 4-oxo-fenretinide, markedly induced G2-M cell cycle arrest and apoptosis in ovarian, breast and neuroblastoma tumor cells [66]. Interestingly, naturally occurring resveratrol, a phytoalexin present in grapes and red wine, potently induced growth inhibition and apoptosis in metastatic breast cancer cells via the activation of SPT and accumulation of ceramide [67].

Recently, it has been shown that the SPTLC2 and SPTLC3 subunits have a different affinity for Acyl-CoA in the function of the chain, giving rise to Cer of different lengths, while SPTLC1 is essential to produce an active enzyme [68,69]. ssSPT subunits have been described as important regulators of SPT activity. Recently, it has been observed that in patients with prostate cancer treated with anti-androgens treatment, an increase in the expression of SPTSSB was detected [70]. Moreover, administration of exogenous Cer nanoliposomes was determined as an efficient treatment in cancer cells [70]. In addition to ssSPT subunits, the Tsc3 subunit was found to be a potent regulatory protein of SPT, increasing its activity by up to 50-fold [71,72].

#### 3.1.2. Ceramide Synthase (CerS)

CerS takes part in both the de novo pathway and the salvage pathway of ceramide synthesis. CerS is a family of six different isoforms, each of which synthesizes ceramides with different chain lengths of fatty acyl-CoA, resulting in a specific activity. This fact is important, since, for example, CerS1 generates 18-carbon Cer (C_18:0_-Ceramide) that inhibits tumor growth [73], whereas CerS5/6 produces C_16:0_-Ceramide, an anti-apoptotic metabolite in human head and neck squamous cell carcinomas [74] that is also involved in type II diabetes [17,33,75] and that has proinflammatory properties [76]. Interestingly, a specific inhibitor of CerS1, called P053, has been described to reduce the levels of C_18:0_-Ceramide in renal HEK 293 cancer cells [77]. Overexpression of CerS2 or CerS4 increases very long chain ceramides (C_24:0_-Ceramide), which have a protective effect on human breast and colon cancer [78]. Recently, increased levels of C_24:0_-Ceramide in gallbladder cancer was observed due to the overexpression of SPT and CerS2 and related to the progression of the tumor [24].

Interestingly, analogs derived from Fingolimod (FTY720) specifically inhibit isoforms of CerS in HCT-106 and HeLa cells. Specifically, the ST1058 and ST1074 inhibitors preferentially inhibit CerS2 and CerS4, whereas ST1072 blocks the activities of CerS4 and CerS6, and ST1060 inhibits CerS2 [79].

It has also been shown that overexpression of CerS in certain types of cancer confers resistance to chemotherapy [44]. Concerning anticancer drugs, anthracyclines have been shown to increase the content of pro-apoptotic ceramides by stimulating CerS [80]. In particular, vinblastine activated CerS, increasing ceramide content in the liver [81]; paclitaxel (taxol) is an antitumor molecule that led to Cer-induced apoptosis in human breast cancer cells by activation of both SPT and CerS [82], and antifolate methotrexate (MTX) increased the levels of C_16:0_-Ceramide to reduce proliferation in a p53-dependet manner in human lung adenocarcinoma cells [83]. In addition, a recent study showed that the activity of CerS6, giving rise to Cer, increases the inhibitory capacity of p53 to block progeny formation in polyploid cancer cells [84]. By contrast, fumonisin B1, a fungal metabolite that potently inhibits CerS, was shown to reduce apoptotic cell death in a variety of cell types, including SCC17B human head and neck squamous carcinoma cells [85].

#### 3.1.3. Dihydroceramide Desaturase (Des1, also Known as DEGS1)

Des1 is the last enzyme in the de novo synthesis pathway of ceramide. This enzyme catalyzes the conversion of dhCer to Cer by insertion of a trans double bond in position 4–5. Experiments using heterozygous DES1-null mice showed higher dhCer/Cer ratios in multiple organs, rendering cells resistant to apoptosis [86]. Furthermore, knockdown of Des1 using siRNA [87] led to cell cycle arrest in human neuroblastoma cells [87]. However, accumulation of dhCer in cells can stimulate autophagy. Although autophagy is a mechanism of cancer cell survival, it can also lead to cell death [88,89,90]. So far, several compounds with the ability to inhibit Des1 have been described. For example, resveratrol, which is a dietary polyphenol with well recognized antioxidant and health beneficial properties, was shown to inhibit Des1, causing autophagy in gastric cancer HGC27 cells [91]. Other Des1 inhibitors, including γ-tocotrienol, phenoxodiol or celecoxib, induce autophagy by dhCer accumulation in T98G and U87MG glioblastoma cell lines by inhibition of Des1 [88]. Furthermore, 3-ketosphinganine and its dideuterated analog at C4 (d2KSa) are metabolized to produce high levels of dihydrosphingolipids (dhSLs) in HGC27, T98G and U87MG cancer cells. d2KSa provokes the accumulation of dhCer and other dhSLs by inhibition of Des1 and induces autophagy in cancer cells [92]. Fenretinide, which is an agonist of SPT, also inhibits Des1 and, combined with Foscan-mediated photodynamic therapy, enhances apoptosis in SCC19 cells, a human head and neck squamous cell carcinoma [93].

N-[(1R,2Shttpo)-2-hydroxy-1-hydroxymethyl-2-(2-tridecyl-1-cyclopropenyl)ethyl]octanamide (GT11) is a specific inhibitor of Des1 that efficiently activates autophagy and apoptosis of the human U87MG glioma cell line. In addition, treatment with tetrahydrocannabinol (THC) produces an alteration of the lipid composition in the endoplasmic reticulum and, along with it, the accumulation of dhCer due to the reduction of Des1 expression, leading to a stimulation of autophagy and apoptosis in human U87MG glioma cells [89]. Moreover, THC treatment or selective inhibition of Des1 by GT11 efficiently activates autophagy and apoptosis and inhibits tumor growth in mice [89].

### 3.2. Enzymes of the Sphingomyelinase Pathway

#### 3.2.1. Sphingomyelinases (SMase)

Based on their optimal pH for activity and their location, there are three major types of SMases: aSMase, nSMase and Alk-SMase. SMases catalyze the breakdown of membrane SM to ceramide and phosphocholine. Stimulation of SMase activity is related to oxidative stress [36], cell death signals such as TNF Receptor (TNFR) and Fas [94,95] or anticancer drugs [96]. Experiments where SMase activity was knocked down showed inhibition of tumor growth in non-small cell lung carcinomas [97]. Furthermore, aSMase-null mice (*smpd1^-/-^*) were protected from pulmonary tumor metastasis [98]. Contrarily, resveratrol was described to inhibit cell proliferation of human leukemia (K562 cell line) and cancer cells (HCT116 cell line) by up-regulation of aSMase and the consequent Cer accumulation [99].

The term FIASMA (Functional Inhibitor of Acid SphingoMyelinAse) encompasses a family of drugs, many of them approved for use in humans, which have been shown to indirectly inhibit the activity of aSMase [100]. Interestingly, recent studies have shown that inhibition of lysosomal SMase via these inhibitors prevents infection of the SARS-CoV-2 virus [101,102,103]. In Niemann–Pick disease, it has been observed that aSMase dysfunction leads to a dysregulation of lysosomes, causing a disruption of cholesterol transport [104]. This process can lead to cell dysfunction and death. Perphenazine and fluphenazine, both FIASMA members, were found to inhibit xenografted tumor growth by disruption of intracellular cholesterol transport [105].

Recently, a small molecule called ARC39 was synthetized and characterized. ARC39 was found to inhibit both lysosomal and secretory aSMase in vitro with L929, HepG2 and B16F10 cells [106]. However, ARC39 inhibition in vivo could not be confirmed; therefore, more studies should be carried out. Moreover, a potent light-inducible photocaged ASM inhibitor (PCAI) with the ability to inhibit aSMase has been developed. Furthermore, being inducible by light, it is addressable [107].

Most studies on the implication of SMase activity in cancer are related to the acidic form of the enzyme, although nSMase may also play an important role in cancer biology [95]. nSMase has also been observed to be involved in the activation and migration of T cells, responsible for the detection and elimination of cancer cells [108]. Doxorubicin is an antibiotic of the anthracycline family widely used in chemotherapy treatment. Interestingly, doxorubicin-induced growth arrest in breast cancer cells is due to a p53-dependent upregulation of nSMase-2 [96]. As seen above, chemotherapy treatments are highly linked to the metabolism of sphingolipids, sometimes in a good way and others in the opposite direction. Recently, it has been observed that doxorubicin treatment stimulated the activity of aSMase, leading to an increase in Cer and reactive oxygen species (ROS), promoting apoptosis [109]. As a side effect, the release of ROS into the bloodstream damaged the vascular endothelium [109]. Exosomes are vesicles between 40–160 nm that can be secreted by a variety of cells. They carry nucleic acids, proteins and metabolites. Their function is that of intercellular communication between nearby and distant cells, both in physiological conditions and in certain diseases. In recent years, their involvement as a biomarker and therapeutic target has gained great interest [110,111]. Exosomes have been found to be crucial for the formation and maintenance of microenvironments in tumors. The need for nSMase activity for the release of exosomes has been described [110,112]. Therefore, certain studies in tumors have focused on the inhibition of this enzyme. In particular, the nSMase isoform, nSMase2, was involved in exosome miRNA secretion and contributed to cancer cell metastasis through the induction of angiogenesis [113]. Specific inhibition of nSMase by GW4869 showed a potential target to block the metastasis. In addition, low expression of nSMase was found in exosomes from hepatocellular carcinoma patients, related with a poor outcome [114]. Similarly, exosomes have been shown to be signaling systems for the immune system, allowing the fight against tumors. Recently, it has been observed that the treatment with platinium nanoparticles enhanced exosomes secretion in A549 lung cancer cells [115].

#### 3.2.2. Sphingomyelin Synthase (SMS)

The sphingomyelin synthase (SMS) family consists of three members, SMS1, SMS2 and SMS-related protein (SMSr) [116]. This enzyme activity catalyzes the synthesis of SM from Cer and phosphatidylcholine, thereby releasing diacylglicerol. It was demonstrated that selective inhibition of SMS by D609 induces an increase in the concentration of ceramide in the ER, and it triggers autophagy in hippocampal neurons [117]. However, in glioblastoma, it was observed that treatment with the 2-hydroxyoleic acid (2OHOA), an anticancer drug, caused an increase in SMS activity. Furthermore, the inhibition with D609 diminishes the anticancer effect [118]. Recently, SMS2 was found to be upregulated in different cancers, such as breast [119] and ovarian [120]. High infiltration of M2-polarized macrophages in primary tumors is related with poor outcomes in patients. Zheng and co-workers demonstrated the disruption of apoptotic pathways by activation of SMS2 in breast cancer [121]. Activation of SMS2 causes a reduction in Cer levels and stimulates cell proliferation through activation of the transforming growth factor-β (TGF-β)/Smad signaling pathway. By contrast, inhibition of SMS2 by specific siRNA led to accumulation of Cer and the promotion of cell death [121]. In addition, recent works observed that SMS2 is upregulated in breast cancer and induces macrophages polarization and tumor progression [119]. Moreover, SMS2 inhibition by 15w or knockdown reduced the release of cytokines that stimulate macrophage polarization, reducing tumor growth [119]. In addition, SMS1 down regulation was found in metastatic melanoma patients and was associated with worse prognosis due to an imbalance between SM and glucosyl-ceramide levels [122].

### 3.3. Enzymes of the Salvage Pathway

#### 3.3.1. Glucosylceramide Synthase (GCS)

Glucosylceramide synthase (GCS) is a lysosomal enzyme that introduces a glycosylation on the ceramide to produce glucosylceramide. GCS has been observed to be upregulated in different types of cancer, being associated with resistance to anticancer treatments [123,124,125,126,127]. Glucosylceramide plasma levels have recently been described as a promised biomarker for prostate cancer status [128]. A recent study has demonstrated that a GCS inhibitor, called 1-phenyl-2-decanoylamino-3-morpholino-1-propanol (PDMP), caused a lysosomal lipid accumulation and inactivation of mTOR in HeLa cells, leading to apoptotic stimulation [129].

#### 3.3.2. Acid β-Glucosidase (β-GCase)

Mutations in the GBA1 gene that codes for β-GCase is one of the main causes of the development of Parkinson’s disease [130,131]. Different studies have associated the deficiency of this gene with the possibility of suffering from certain types of cancer [132,133]. Recently, it was reported that β-GCase was overexpressed in chemotherapy-resistant gastric cancer cells [134]. In addition, it was observed that its inhibition with specific siRNAs produced a lysosomal dysfunction that, together with anticancer treatment, caused cell death [134].

#### 3.3.3. Ceramidase

As seen above, ceramidases catalyze the degradation of ceramide to sphingosine. Ceramidases have been described as important regulators of the processes of cellular autophagy and resistance to chemotherapy. Therefore, inhibition of the different ceramidase isoforms has been shown to be beneficial for the treatment of different types of cancer [49,52,135]. In recent years, different inhibitors have been developed that have shown their potential as an anticancer therapy [136].

Acid ceramidase (ASAH1) has been detected to be overexpressed in melanomas, conferring resistance to chemotherapy. The involvement of ASAH1 in mitochondrial function and cellular autophagy in melanoma cells has been observed [137]. Furthermore, a recent study has shown that deletion of the ASAH1 gene blocks the induction of apoptosis mediated by doxorubicin [138]. These results may be due to the high adaptive capacity of melanoma cells. However, inhibition of ASAH1 with siRNA or pharmacologically with LCL521 in colorectal cancer cell lines (HT29, HCT116 and LIM1215) enhanced X-ray radiosensitivity [139]. Moreover, tamoxifen has shown its ability to inhibit ASAH1 in polyploid giant cancer cells by reducing their division [140]. Recently, the synthesis of N-metallocenoylsphingosines has been described, demonstrating its cytotoxic capacity and inhibition of the cell cycle in cancer cells [141].

Alkaline ceramidase (ACER) has been described as being highly related to the growth, migration and invasion of tumor cells [142,143,144]. In this sense, incubation of HepG2 and Huh-7 human liver cancer cells with the ACER2 inhibitor, called D-erythro-MAPP (D-e-MAPP), has been shown to reduce cell growth [142]. Structural analogues of ceramides have demonstrated their efficacy as selective ACER inhibitors, blocking cell growth and being suitable candidates for anticancer treatment [145,146]. More specifically, deoxy-sphingolipid analogs have recently been described as highly selective molecules and potent ACER3 inhibitors, with the ability to inhibit the cell cycle [147]. Of interest, the structure of ACER3 has recently been described, allowing structural and computational studies for the development of new specific inhibitors [148].

Neutral ceramidase is mostly expressed in the intestine and is highly related to the progression of colon cancer [149]. Studies with ASAH2 knockdown (ASAH2^-/-^) mice demonstrated that deletion of ASAH2 inhibited cell and tumor growth, both in vitro and in vivo, through the WNT/β-catenin, ERK, GSK-3β and Akt pathways [150,151]. In addition, inhibition with a specific inhibitor, C_6_-urea-ceramide, obtained similar results [150,151].

Adiponectin receptors (ADIPORs) are membrane proteins implicated in the control of glucose and lipid metabolism. Different studies have determined the incidence relationship of certain cancers with polymorphisms of the genes that code for ADIPORs [152,153,154]. Moreover, several studies have demonstrated the efficiency of ADIPORs agonists to induce cell death in cancer cells, such as (2-(4-Benzoylphenoxy)-N-[1-(phenylmethyl)-4-piperidinyl]-acetamide) in cholangiocarcinoma [155], ADP355 in prostate cancer [156] or AdipoRon with pancreatic cancer cells [157]. Interestingly, it has recently been described that ADIPORs have ceramidase activity in their intracellular domain, increasing the levels of Cer, a pro-apoptotic metabolite [158].

### 3.4. Sphingosine Kinase (SphK)/Sphingosine 1-Phosphate Phosphate (SPP)

#### 3.4.1. Sphingosine Kinase (SphK)

S1P is one of the most studied bioactive sphingolipids in cell signaling and metabolism. It was first described as a pro-mitogenic and anti-apoptotic molecule [159]. The enzymes responsible for S1P generation are the sphingosine kinases (SphK), of which two different isoforms have been described, SphK1 and SphK2 [7,8]. In addition to its mitogenic, antiapoptotic and proinflammatory actions [4,160], S1P is implicated in cell migration, differentiation or tumorigenesis, and it is a key regulator of angiogenesis [161,162,163,164,165,166,167,168,169,170,171]. Besides being generated intracellularly, S1P is also present in the plasma at relatively high concentrations (0.1 µM to 1 µM) [172]. Interestingly, plasma S1P has been associated with its chemoattractant and pro-metastatic actions in cancer cells [162,171]. S1P is particularly increased in the plasma of patients with lung cancers [18] but has been implicated in the dissemination of tumors other than lung cancer, such as melanoma, human leukemia and hepatocellular carcinoma (HCC) [171,173,174,175,176].

A recent study in healthy volunteers revealed that S1P is also a major peripheral blood chemoattractant for bone marrow (BM)-residing hematopoietic stem/progenitor cells [177]. An interesting aspect of SphKs is their topology; whereas SphK1 is located in the cytosol, SphK2 is present in the nucleus. The different location of the two SphKs appears to be responsible for their different roles in cellular processes. Both SphK1 and SphK2 are related to inflammatory processes, cell proliferation and tumor spreading [178]. 

Overexpression of SphK2 has been related to cell growth arrest and apoptosis stimulation [179,180,181]. However, its overexpression was found in the nuclei of MCF7 human breast cancer cells [182] and was found to promote osteosarcoma cell growth [183]. 

Interestingly, in a clinical study with patients with glioblastoma, a correlation between SphK1 and poor survival was observed [184]. Moreover, selective inhibition of SphK1 or SphK2 with siRNA in U-1242 and U-87MG glioblastoma cell lines showed an inhibition of cell proliferation, with more potent action when SphK2 was silenced [184]. In addition, SphK2 has been implicated in chemotherapeutic drug doxorubicin-mediated apoptosis in HCT116 colon carcinoma cells, increasing its effect by down-regulation of SphK2 with specific siRNA [182]. More recently, it has been observed that a specific SphK2 inhibitor (ABC294640) increases the antitumor effect of tumor necrosis factor-related apoptosis inducing ligand (TRAIL) treatment in non-small cell lung cancer [185]. 

Another important feature of S1P biology has been the development of specific inhibitors of SphK1, as well as agonists and antagonists of S1P itself. These compounds have shown efficiency and effectiveness in the treatment of a variety of diseases, including liver fibrosis, multiple sclerosis or cancer. Specifically, fingolimod (FTY720) is an important S1P receptor antagonist used in the clinic [186,187,188,189]. This compound has been used to treat a variety of inflammatory diseases, including multiple sclerosis [190,191] or uveoretinitis [192], and can reduce liver and lung metastasis in rats [171]. However, due to its immunosuppressant properties, fingolimod might increase the risk for developing other types of cancer and might facilitate opportunistic infections that may have detrimental effects or be fatal for the organism [193]. In addition, down-regulation of SphK1 by specific siRNA or the specific inhibitor SK1-I (also referred to as BML-258) induces apoptosis and suppresses growth of human glioblastoma cells and xenografts by a process involving inhibition of protein kinase B (PKB, also known as Akt) and the activation of c-Jun N-terminal kinase (JNK) [194]. Another selective SphK1 inhibitor, N,N-Dimethyl-D-erythro-sphingosine (DMS), was shown to modulate cellular ceramide levels and induce apoptosis in the human lung cancer cells [195]. In addition, it has been described how the co-treatment of two SphK1 inhibitors, FTY-720 and PF453, together with doxorubicin, inhibits the cell growth of breast cancer cells [196]. Moreover, in other recent work, it was observed that the Kirsten rat sarcoma viral oncogene homolog (K-Ras) produced an increase in S1P and Cer levels. By SphK1 inhibition with PF453 inhibitor, or by silencing its expression, a decrease in S1P levels was determined, whereas no change in Cer levels were observed, triggering the block of the cell cycle [197].

There is also evidence showing that cancer cells stimulate the release of S1P from neighboring cells to self-stimulate cell proliferation and migration. Of interest, extracellular S1P exerts its biological functions through interaction with five different G-protein coupled receptors, named S1P receptors 1 to 5 (S1PR1-5). For details on the biology of S1P receptors, the reader is referred to elegant reviews by A. Carter et al. [198], M. Maceyka et al. [170] or N.J. Pyne et al. [199].

#### 3.4.2. Sphingosine 1-Phosphate Phosphatase (SPP, also Known as SGPP)

Sphingosine and S1P are readily interconvertible by the action of specific kinases and phosphatases (SPP1 and SPP2) [200] and, possibly, also by non-specific extracellular lipid phosphate phosphatases (LPPs) [55,201]. These phosphatases hydrolyze the phosphate group of S1P to produce sphingosine, which can then be converted to ceramide by CerS [202]. In animal models lacking expression of SPP1 and SPP2, developmental disorders in the epidermis and gastric tract have been observed [203,204], suggesting a relevant role of these enzymes in epithelial and skin integrity and development. In addition, mouse models with the deletion of exons 3 and 4 of the genes coding for SPP1 and SPP2 produced mouse neonates that died within a few days or adult mice that developed type 2 diabetes, respectively [205]. Furthermore, SPP1 expression was significantly lower in gastric tumor tissue than that in normal tissue, and treatment with specific SPP1 siRNA showed increased cell migration and invasion in gastric cancer [206]. Noteworthily, silencing of SPP1 in epidermal cells increased the chemoattractant effect of epidermal growth factor (EGF) [207], and overexpression of microRNA miR-27a reduced SPP1 levels [208], leading to a promotion of colorectal cancer. For details on the role of SphK and SPP in cancer biology, the reader is referred to elegant reviews by L.M. Obeid and coworkers [209,210] and N. Pyne and coworkers [199,211].

### 3.5. Ceramide Kinase (Cerk), Phosphatases and CERT

C1P was first demonstrated to be mitogenic [212,213,214,215,216] and to inhibit apoptosis in a variety of cell types [217,218,219,220,221], thereby implicating CerK in the regulation of cell growth and survival. The role of CerK in cell growth and survival of normal and transformed cells has been widely addressed by different groups (reviewed in [4,222]). The mechanisms whereby C1P exerts its mitogenic or pro-survival actions include activation of different signaling pathways, such as mitogen-activated protein kinase kinase (MEK), extracellularly regulated kinases (ERKs) 1/2, phosphatidylinositol 3-kinase (PI3K)/Akt, mammalian target of rapamycin (mTOR), c-Jun N-terminal kinase (JNK) and protein kinase C-α [6,189,215,223], or stimulation of vascular endothelial cell growth factor (VEGF) secretion [216]. C1P-promoted cell survival involved direct inhibition of aSMase [224,225] or SPT [220], upregulation of inducible nitric oxide synthase (iNOS) expression [219] or activation of Akt [218]. CerK is particularly relevant in lung and breast cancer cell proliferation and dissemination [226,227]. Down-regulation of CerK expression using specific siRNA blocked progression of A549 human lung adenocarcinoma cells, reduced cancer cell proliferation and enhanced apoptosis in these cells [226]. Likewise, inhibition of CerK activity with the selective inhibitor NVP-231 blocked human epithelial lung cancer cell proliferation [228], whereas overexpression of CerK enhanced cell proliferation and protected against apoptosis. Furthermore, upregulation of CerK is profoundly important for the development of human breast cancer. Studies from patient biopsies revealed that increased CerK expression is associated with an elevated risk of tumor recurrence in women with breast cancer [229].

Another relevant aspect of Cerk biology is its implication in inflammatory responses. Initial studies by Chalfant and co-workers demonstrated the proinflammatory properties of C1P in different cell types (reviewed in [230,231,232]). It should be noted that chronic inflammation is closely associated with tumor development of different types of cancer. However, in normal lung cells, C1P seems to exert anti-inflammatory actions [233,234,235]. The dual actions of C1P in inflammation have been previously reviewed [4,222,236].

Several drug-resistance cancer cell lines showed upregulated levels of CERT. Recently, the effectiveness of the inhibition of Cer transport to the Golgi apparatus by inhibition of CERT has been demonstrated as an anti-cancer treatment [237]. The HPA-12 inhibitor had already demonstrated its effectiveness as a CERT inhibitor, increasing Cer concentration and cell death [238]. Since then, several studies have described structurally similar inhibitors, effective in inhibiting CERT [239,240]. Recently, Nakao et al. synthesized and characterized a new CERT inhibitor, named (1S, 2R)-HPCB-5 [241]. Moreover, structural and affinity studies, followed for binding experiments of Cer to CERT, have been carried out on different drugs. This study has shown that drugs such as Lomitapide, Clevidipine, Fluralaner and Eltrombopag inhibit CERT in HeLa cells [242]. In addition, N,O-Dialkyl deoxynojirimycin derivatives have been described as CERT inhibitors in vitro [243].

Inhibitors and activators of enzymes involved in sphingosine and ceramide metabolism are summarized in Table 1.

## 4. Concluding Remarks

Regulation of sphingolipid metabolism enzymes is a major topic of study in cell biology and disease. Both intracellular and extracellular sphingolipid levels are tightly controlled to ensure proper cell and tissue homeostasis. Ceramides are tumor suppressors; distinct molecular species may be implicated in different cellular functions, and they likely reflect distinct pathophysiological states. The above discussions point to ceramide metabolism as a potential target for developing novel strategies to treat tumor promotion and dissemination. Additionally, it was found that the combined use of conventional drugs with effectors of ceramide metabolism would make it possible to improve the effectiveness of conventional therapies.

The synthesis of new inhibitors of sphingolipid metabolism has shown its effectiveness. It has been observed that, depending on the type of cancer, the expression of sphingolipid metabolism enzymes is altered. The study of these variations, as well as the discovery of new inhibitors, is of vital importance.

## Figures and Tables

**Table 1:** Enzyme inhibitors and activators that efficiently alter ceramide metabolism.

Targeted Enzyme	Effector (Function)	Tissue/Cell Type	Ref.
**SPT**	Myriocin (inhibitor)	B16F10 melanoma cells; Human lung adenocarcinoma cell line (HCC4006); Human glioblastoma cells (U87MG) Breast cancer cells	[19,61,62,63]
	Tetrahydropyrazolopyridine; 3-phenylpiperidine (inhibitors)	PL-21 cells derived from an acute promyelocytic leukemia; Human lung adenocarcinoma cell line (HCC4006)	[27,64]
	Fenretinide (activator)	Ovarian; Breast and neuroblastoma tumor cells	[65,66]
	Taxol (activator)	Human breast cancer cells	[82]
	Resveratrol (activator)	Metastatic breast cancer cells	[67]
	Anti-androgens (activator)	Prostate cancer	[70]
**CerS**	Anthracyclines, Vinblastine (Activators)	Liver	[80,81]
	Taxol (activator)	Human breast cancer cells	[82]
	Antifolate methotrexate (indirect activator)	A549 lung adenocarcinoma cells	[83]
	P053 (inhibitor)	HEK 293 cells	[77]
	ST1058, ST1060, ST1072, ST1074	HCT-106 and HeLa cells	[79]
**Des1**	Resveratrol (inhibitor)	Gastric cancer HGC27 cells; T98G and U87MG glioblastoma cell lines	[88,91]
	γ-tocotrienol, phenoxodiol, celecoxib (inhibitors)	T98G and U87MG glioblastoma cell lines	[88]
	Dideuterated analog of 3-keotsphinganine (inhibitor)	HGC27, T98G and U87MG cancer cells	[92]
	Fenretinide combined with Foscan (inhibitor)	SCC19 human head and neck squamous cell carcinoma	[93]
	GT11 (inhibitor)	Human U87MG glioma cell line	[89]
	Tetrahydrocannabinol (THC) by indirect mechanism inhibition		
**SMase**	Gamma radiation (activator)	Liver	[36]
	GW4869 (inhibitor)	Lung from human melanoma cells	[113]
	siRNA (inhibitor)	Adenocarcinoma cell line A549	[97]
	Doxorubicin (activator)	Breast cancer cells	[96]
	Resveratrol (activator)	K562 and HCT116 cell lines	[99]
	Perphenazine and fluphenazine (inhibitor)	Xenografted tumor growth	[105]
	ARC39 (inhibitor)	L929, HepG2 and B16F10 cell lines	[106]
**GCS**	PDMP	HeLa cell line	[129]
**β-GCase**	siRNA	Gastric cancer	[134]
**Ceramidase**	LCL521 (inhibitor)	HT29, HCT116 and LIM1215 cell lines	[139]
	siRNA		
	Tamoxifen (inhibitor)	Polyploid giant cancer cells	[140,244]
	N-metallocenoylsphingosine (inhibitor)	High-5 insect cells	[141]
	D-erythro-MAPP (inhibitor)	Hepatocellular carcinoma cells	[142]
	Deoxy-sphingolipids (inhibitor)	Several cancer cells	[145,147]
	C_6_-urea-ceramide (inhibitor)	Colon cancer cells	[150,151]
**SMS**	D609 (inhibitor)	Hippocampal neurons and glioblastoma	[117,118]
	SMS siRNA	Breast cancer	[119,121]
	15w (inhibitor)		[119]
**SphK1**	SK1 siRNA	U-1242 and U-87MG glioblastoma cell lines	[184]
	SK1-I (BML-258) (inhibitor)	Human glioblastoma cells and xenografts	[194]
	FTY-720 (Degradation inhibition)	Lymphocytes, microglia, myeloma, leukaemia, prostate, lung, liver, breast, colon, gastric, bladder, renal glioma and ovarian cancers	[186,187,245,246,247,248,249,250,251,252,253,254,255,256,257]
	SphK^-/-^ mouse models	Murine melanoma cells	[173]
	N, N-Dimethyl-D-erythro-sphingosine (DMS) (inhibitor)	Human lung cancer	[195]
	FTY-720 and PF453	Breast cancer cells	[196]
	PF453	MCF10A cell line	[197]
	SK1-II	Liver cancer cells	[258]
**SphK2**	Sphk2 siRNA	U-1242 and U-87MG glioblastoma cell lines and HCT116 colon carcinoma cells	[182,184]
	ABC294640 (inhibitor)	non-small cell lung cancer	[185]
**SPP**	miR95 and miR21(inhibition)	Lung cancer	[259,260]
	miR-27a (inhibition)	Colorectal cancer	[208]
**CerK**	siRNA	A549 human lung adenocarcinoma cells	[226]
	NVP-231 (inhibitor)	MCF-7 and NCI-H358 human lung cancer cell lines	[228]
**CERT**	HPA-12 family (inhibitors)	Chinese hamster ovary cells, HeLa cells	[238,239,240]
	(1S, 2R)-HPCB-5	HeLa cells	[241]
	Lomitapide, Clevidipine, Fluralaner and Eltrombopag (inhibitors)	HeLa cells	[242]
	N,O-Dialkyl deoxynojirimycin	in vitro	[243]
